# Systematic Review on the Impact of Guidelines Adherence on Antibiotic Prescription in Respiratory Infections

**DOI:** 10.3390/antibiotics9090546

**Published:** 2020-08-27

**Authors:** Inês Oliveira, Catarina Rego, Guilherme Semedo, Daniel Gomes, Adolfo Figueiras, Fátima Roque, Maria Teresa Herdeiro

**Affiliations:** 1 Faculty of Health, Medicine and Life Sciences, University of Maastricht, 6200 MD Maastricht, The Netherlands; inesofia17@hotmail.com; 2 Faculty of Pharmacy of the University of Lisbon, 1649 Lisbon, Portugal; catarinarego@ua.pt; 3 Department of Medical Sciences, University of Aveiro, 3810 Aveiro, Portugal; guilhermesemedo@ua.pt; 4 Research Unit for Inland Development, Polytechnic of Guarda (UDI-IPG), 6300 Guarda, Portugal; danielsgomes98@outlook.pt; 5 Department of Preventive Medicine and Public Health, University of Santiago de Compostela, 15702 Santiago de Compostela, Spain; adolfo.figueiras@usc.es; 6 Health Research Institute of Santiago de Compostela (IDIS), 15706 Santiago de Compostela, Spain; 7 Consortium for Biomedical Research in Epidemiology & Public Health (CIBERESP), 28001 Madrid, Spain; 8 Health Sciences Research Centre, University of Beira Interior (CICS-UBI), 6200 Covilhã, Portugal; 9 Department of Medical Sciences, Institute of Biomedicine–iBiMED, University of Aveiro, 3810 Aveiro, Portugal; teresaherdeiro@ua.pt

**Keywords:** review, respiratory tract infections, guidelines, adherence, antibiotic prescription

## Abstract

Overuse and inappropriate antibiotic prescription for respiratory tract infections (RTI) are one of the major contributors to the current antibiotic resistance problem. Guidelines provide support to prescribers for proper decision-making. Our purpose is to review the impact of prescribers’ exposure to guidelines in antibiotic prescription for RTIs. A systematic review was performed searching in the scientific databases MEDLINE PubMed and EMBASE for studies which exposed prescribers to guidelines for RTI and compared antibiotic prescription rates/quality before and after the implementation, with thirty-four articles included in the review. The selected studies consisted on a simple intervention in the form of guideline implementation while others involved multifaceted interventions, and varied in population, designs, and settings. Prescription rate was shown to be reduced in the majority of the studies, along with an improvement in appropriateness, defined mainly by the prescription of narrow-spectrum rather than broad-spectrum antibiotics. Intending to ascertain if this implementation could decrease prescription costs, 7 articles accessed it, of which 6 showed the intended reduction. Overall interventions to improve guidelines adherence can be effective in reducing antibiotic prescriptions and inappropriate antibiotic selection for RTIs, supporting the importance of implementing guidelines in order to decrease the high levels of antibiotic prescriptions, and consequently reduce antimicrobial resistance.

## 1. Introduction

Antibiotic resistance is an important public health issue and one of the World Health Organization’s [WHO] highest priorities [1]. As a consequence of the growing levels of antibiotic resistance, higher patient morbidity and mortality rates have been registered, along with an increase in healthcare expenditure and several guidelines adjustments over the last decades [2,3]. Worldwide, the number of deaths could reach 700,000. Moreover, in Europe, it is estimated that multidrug-resistant bacteria are annually responsible for 25,000 deaths and 1.5 billion euros spent in extra healthcare costs and productivity losses. Furthermore, if measures are not taken to reverse current resistance rates, it is expected that by 2050, 2.9 trillion USD will be spent in cumulative losses in OECD (Organization for Economic Co-operation and Development) countries and that 10 million deaths will occur per year. Of this value, Europe is expected to have 392k deaths and Asia and Africa to be the continents with the highest number (4.7M and 4.1M, respectively) [4].

Inappropriate antibiotic prescription and overuse are the most preponderant contributors to the current antibiotic resistance crisis, with respiratory tract infections (RTIs) responsible for the majority of antibiotic prescriptions [5,6,7,8,9,10,11]. Although most RTI’s are self-limiting, and the effects of antimicrobial treatment for RTI’s have been studied and shown to only have a marginal effect in preventing complications, they still represent close to 60% of antibiotic prescriptions in primary care [12,13,14]. Studies have shown that these high rates of improper prescribing are highly influenced by patient expectation, time pressure, but also uncertainty about the best clinical procedure [15,16]. Therefore, healthcare professionals play an important role in tackling this problem. Several clinical interventions have been carried out in order to improve prescription’s quality and to reduce antibiotic misuse in general and, particularly, in RTIs [17,18,19,20]. Some of the interventions implemented involved the presentation or clarification of antibiotic prescription guidelines [17,18]. These guidelines provide support for proper decision-making based on evidence regarding management, diagnosis, and practical strategies for prescribing antibiotics, advising on whether or not a patient needs antimicrobial treatment and which antibiotic should be prescribed [21,22].

Even though systematic reviews regarding the impact of interventions in antibiotic prescription for RTIs have already been published, the impact of the prescriber’s guideline’s adherence remains to be addressed [23,24]. This systematic review aims to close this gap by enlightening and summarizing the effectiveness of prescribers’ exposure to guidelines in antibiotic prescription for RTIs.

## 2. Results

### 2.1. Selection of Papers

**Figure 1:**
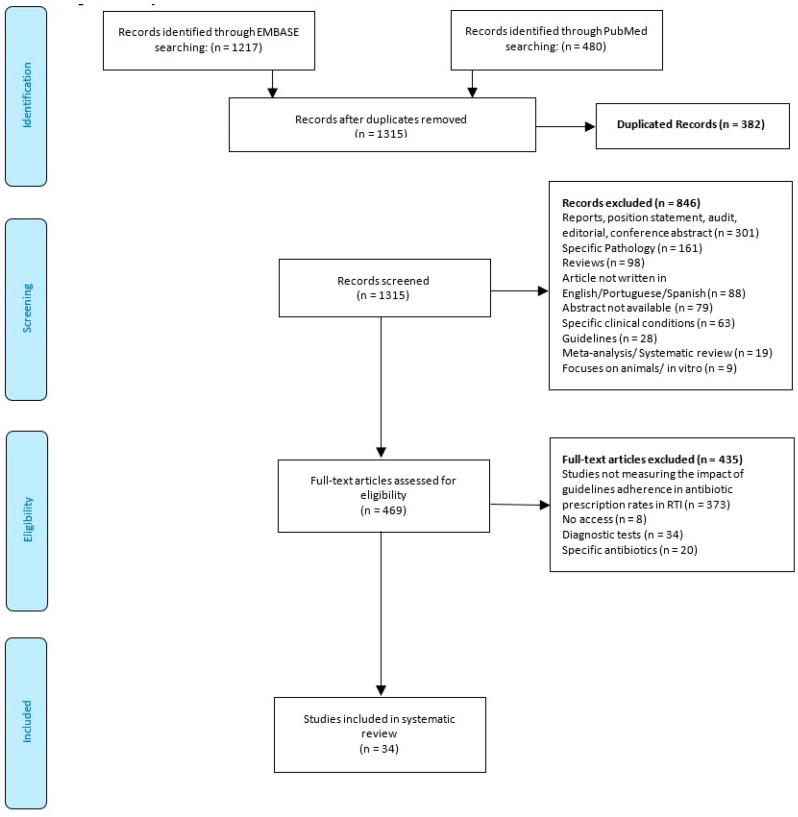
PRISMA (Preferred Reporting Items for Systematic Reviews and Meta-Analyses) flowchart.

The search of the databases yielded 1315 citations, once duplicates had been removed (Figure 1). After screening titles and abstracts, 469 articles potentially met the inclusion criteria. From the analysis of their full text, 435 articles were excluded, and 34 studies were included in this systematic review [25,26,27,28,29,30,31,32,33,34,35,36,37,38,39,40,41,42,43,44,45,46,47,48,49,50,51,52,53,54,55,56,57,58].

### 2.2. Study Characteristics

A description of the characteristics of the included studies is presented in Table 1. Among the included studies, 2 were conducted in Australia [25,44], 14 in Europe (including Israel) [29,30,33,34,38,39,41,42,43,45,47,48,51,55], 8 in North America (including Mexico) [31,32,35,36,40,46,56,57], 1 in South America (Bolivia) [53], and 8 in Asia [26,28,37,49,50,52,54,58]. One study was conducted in Europe and Argentina [27].

Regarding the setting where the guidelines were implemented, 4 were implemented exclusively in hospital care [25,26,30,40], 22 in primary care [27,28,31,32,33,35,37,38,41,42,43,45,47,48,49,51,52,53,54,55,56,57], 3 in nursing homes [36,39,46], 2 in medical training facilities [44,50], and 1 in ambulatory care [58]. Two studies were conducted both in hospital care and primary care [29,34]. The majority of the studies consisted on guideline implementation targeting physicians, with only 1 study targeting other prescribers (nurses), in a primary care setting [37]. With respect to the type of patient in which the studies focused, 10 looked into pediatric [25,29,30,31,34,37,45,48,49,50], 5 only focused in adults [26,35,41,52,55], 2 in elderly [39,46], and the majority included all age groups [27,28,32,33,38,40,42,43,44,47,51,53,54,58], while one article did not define the patient type [57].

Regarding study design, 9 studies were randomized controlled trials [25,31,33,35,37,41,42,49,52], with 5 of these being clustered [31,33,41,49,52]. Two studies were non randomized controlled trials [43,44]. Five studies were quasi-experimental [36,38,39,45,46], one of them also defined as an interrupted time series [45]. There were 3 observational trials [29,30,34], one was retrospective [29], and one study was prospective [34]. Five studies were simply classified as before and after studies [48,50,55,56,57], and only 3 of them were controlled [55,56,57]. However, some studies have not classified their design, so they were left as non-defined [26,27,28,32,40,46,47,51,53,54,58].

The majority of the studies included acute respiratory tract infections as the clinical condition [25,29,31,32,33,35,37,43,45,50,56], with 5 studies focusing only in upper respiratory tract infections [26,30,44,49,58]. One study regarding ARTI (acute respiratory tract infections) mentions that the guidelines implemented are focused on URTI (upper respiratory tract infections) [34].

In relation to the data source, 15 articles collected information through medical records [26,29,31,33,34,35,37,40,43,44,45,48,49,50,52], 10 articles from medical records obtained from surveys [27,28,30,32,36,37,39,51,53,54,58], and 3 articles through health insurance reports [25,42,55]. One article obtained the information from only pharmacy records [41] and one from nursing home records [46]. However, 3 articles obtain information through two sources, namely, one through pharmacists’ claims and health insurance [57], another through pharmacy records and medical records [38] and, finally, one through interviews and medical records [56].

Finally, the type of guidelines implemented were identified as local, national, or international. However, this identification was not possible with the information provided in 3 studies [35,46,49].

### 2.3. Quality Assessment

As shown in Table 1, five studies were evaluated as having a moderate methodological quality, while the remained studies were rated as methodologically weak. No study was rated as methodologically strong. The most common limitations identified included the absence of indication about confounders control [25,28,29,30,31,32,35,36,37,39,43,44,45,46,47,48,49,51,52,53,54,55,57,58] and the lack of information about blinding [25,26,27,29,31,32,33,34,35,36,39,40,41,42,45,48,49,50,51,53,54,55,56,57,58].

### 2.4. Study Outcomes

#### 2.4.1. Description of the Guidelines Implementation

The interventions of the included studies are summarized in the Table 2. In the articles included, the evaluation of the effect of guidelines implementation in prescribing behavior could be the main focus [25,26,28,29,30,31,34,35,37,38,40,43,45,48,50,52,53,54], or implementation could be a part of a broader intervention for prescribers in order to improve the quality and/or the reduction of antibiotic prescriptions. Within these interventions, some studies are associated with multifaceted interventions [33,41,44,55,56,58], as is the case of the HAPPY AUDIT study [27,47], involving a wider population; and tailored interventions, quality improvement [32,36,46], and stewardship programs [49].

From the studies focusing only on guideline implementation, 4 implemented the Practical Approach to Lung Health (PAL) guidelines [28,52,53,54], and in 1 study, the guidelines were applied by the nudging of the prescribers [35]. Regarding the PAL guidelines, although these are international, the 4 studies adapted these guidelines nationally, as shown in Table 1. Except for the cohort studies included in this study, the remaining studies implemented the guidelines through educational interventions.

The majority of the studies consisted on an intervention lasting from a few weeks up to three years, whose effect was compared to a baseline. There was one particular study with a longer analysis period and consisted on a five years’ baseline period and a five years’ post-intervention period [38]. In some cases, there were also studies with no baseline identified [27,34,40]. Some studies compared small similar periods of different years [29,32,35,36,41,54] or within the same year [31]. Only ten studies also performed a follow up analysis [29,31,32,33,36,41,48,51,54,55].

#### 2.4.2. Rates and Quantity of Antibiotics Prescribed for Patients with RTI

The quantitative evaluation of the effectiveness of guidelines implementation was reported in twenty-two studies [25,26,27,28,29,30,32,33,34,36,37,41,42,44,45,46,48,49,52,53,54,55]. The outcomes were reported in a broader RTI, however some studies specified for pathology [27,29,30,33,41,44,52], age [44], and class of antibiotic [34].

The reduction of antibiotic prescriptions was significant in the majority of studies [25,28,29,30,32,34,36,37,41,42,45,48,49,54,58] and other studies had significant reductions but not for all variables. In Bjerrum et al. [27], the reduction was significant in all countries where the intervention was implemented except for Denmark and Sweden; in Magin et al. [44], this reduction is only significant for acute bronquitis/bronquiolitis, but not for URTI. Four studies did not report a significant reduction for any variable [46,52,53,55], with two regarding PAL guidelines. Shrerta et al. [46] was one of these studies, but only pneumonia was a variable of interest for this analysis. Except for Hingorani et al. [40], the remaining studies focusing on URTI assessed this outcome, with Camacho et al. [53] being the only one not reporting significant reduction on antibiotic prescription.

#### 2.4.3. Rates of Appropriate Antibiotics Prescribed for Patients with RTI

The outcome regarding the change in rate of appropriate antibiotic prescription was quantitatively evaluated in eighteen of the selected studies. From these studies, none revealed a decrease in appropriate prescriptions, and only three studies reported no statistically significant change [39,52,55]. Although all other studies reflected an increase in appropriate prescribing behavior, four of them did not specified which were the changes in antibiotic prescription that reflected this improvement [35,40,49,56]. A characteristic present in several studies is the description of appropriate behavior as the use of narrow-spectrum rather than broad-spectrum antibiotics [26,31,33,43,45,47]. Some studies, however, only specify that the increase in appropriate behavior results from a decreased use of broad-spectrum antibiotics [31,45]. While others specify, the occurrence of an increase in prescription of penicillin V, the recommended narrow-spectrum antibiotic, accompanied by a decreased use of other antibiotics [26,33,43,47]. In a similar pattern, another study showed an increase in amoxicillin prescription, also narrow-spectrum, accompanied by a decrease in amoxicillin/clavulanate and in cefpodoxime [34].

There were a few studies which had more in depth descriptions of the results. Rautakorpi et al. [51] describes an increase in prescriptions following guideline recommendations not only regarding the choice of antibiotic type, but also in the choice of therapy duration. Another study describes a decrease in amoxicillin/clavulanate and macrolide prescriptions as the particular changes generating the improved appropriate prescribing behavior [41]. The study published by Aoybamroong et al. [50] shows a decrease in inappropriate prescriptions, particularly for common cold diagnosis and regarding the use of amoxicillin. There was a study that, despite showing a general improvement in appropriateness, also presented some contradictory results, since for beta-lactams, there was indeed an improvement, while for macrolides, an increase in inappropriate prescriptions was registered [38].

One study was particularly interesting as it accessed antibiotic appropriateness based on reports of prescriptions claims at a pharmacy rather than on reports from the prescribing entity [57]. In this particular study, it was observed that after an intervention, there was an increase on appropriate prescriptions, shown trough the higher likelihood of prescribing the recommended first-line rather than second-line antibiotics, caused mostly by a reduction in the number of prescriptions for second-line antibiotics [57].

#### 2.4.4. Changes in RTI Treatment Related Costs

As a secondary outcome, this review aimed to determine if the implementation of guidelines could be effective in reducing the cost related to RTI treatment. Particularly, the focus was to look into changes on the costs related to antibiotic prescription, however, some studies look into the general change in treatment costs, then making a divide into the costs related to antibiotic and non-antibiotic prescriptions [28,37,50]. In order to evaluate this outcome, studies which looked into the cost generated by prescribing antibiotics were selected. Seven from the included articles studied this outcome [28,37,42,49,52,53,54], including all the four studies regarding the PAL guidelines [28,52,53,54], and all studies were carried out in a primary care setting. Within these four studies, three of them [28,53,54] were successful in reducing antibiotic prescription cost for RTI with these specific guideline implementations, with Sherestha et al. [52] not reporting significant improvements.

From the remaining three studies, two have shown significant reduction in cost prescription [37,49], both in pediatric primary care, with Ferrat et al. [42] not presenting significant reduction in cost prescription. Within these last three, Wei et al. [49] was the only one assessing this outcome with follow-up, with significant improvements for the intervention period and follow up, comparing with the baseline values. Opposing to the other outcomes, a variety of settings were described, all studies related to cost analysis were set in primary care.

## 3. Discussion

Prescribing accordingly to guidelines intends to reduce antibiotic prescriptions by not prescribing in consultations where antibiotics are not the appropriate line of treatment, as well as to an improvement in quality, by prescribing the first line antibiotic for the respiratory disease correctly diagnosed. Overall evidence suggests that guideline implementation was effective in reducing antibiotic prescriptions, either as the main focus or associated to a broader intervention. In the articles that did not obtain significant reductions, these were justified by lack of adherence to the guidelines, small sample size, and lack of monitoring of the intervention, factors plausible to interfere negatively in the intervention. Even though these rates were measured in different ways, in different populations and settings, in general, these implementations had the ability to reduce antibiotic prescriptions. Nevertheless, a significant reduction is not a synonym of achieving the desirable appropriate prescription rate, and so it is important to persist on this issue. Regardless of the selected method, the implementation of a continuing rather than a onetime intervention is important in sustaining the effect [46,48,49]. However, evidence for this is lacking as there is a reduced number of studies which check the long term effects of the intervention [28,31,39,44,52]. The majority of the studies done thus far cannot predict the long term effects of the intervention [34].

Interestingly, a pattern was noticed when the study accessed the rates of antibiotic prescribing and its appropriateness, as both had the same significant result, suggesting that reducing inappropriate prescriptions is directly related with reduction of the antibiotic prescription rates, providing more information towards this theory discussed in previous reviews [18].

Predominantly, there was a common increase in appropriate prescribing behavior as a result of the intervention. Although several studies make it a point to mention that despite this improvement, values remain high and further changes are necessary [26,30,35,47,48].

From the evaluated studies, some focus on the effect of guideline implementation, rather than of a direct intervention, whilst also mentioning the necessity for further studies supporting guideline effectiveness [26]. Dissemination of these guidelines has been used on several occasions to improve appropriateness, and some effect, although small, has been shown, proving their importance [28,29,34,37,45,54]. However, there are also reports were their implementation failed to achieve a change [51,52,53]. This could correlate to existence of an already strong percentage of appropriate prescribing practices at baseline, generating a ceiling effect [44,51].

Overall, the outcomes obtained also show that an educational intervention focused on adherence to the guidelines is sufficient to enhance the quality of the prescriptions and their quantity. So, despite the occurrence of cases being simple interventions, such as guideline implementation or the use of written information to spread awareness of inappropriate prescribing practice, they have led to an improvement in appropriateness, it is commonly mentioned that the choice of the method for dissemination of knowledge is important in improving results, since studies covering other actors such as patients are also essential to raise awareness about this problem and to promote good behaviors towards the use of antibiotics [25,26,32,51,59,60]. Evaluated studies describe that a more complex intervention is necessary for an effective change in behavior [25,38,53,61,62,63]. By complex intervention, it is meant that several approaches are used simultaneously to educate and alter prescribing practices, and particularly, more direct interventions like workshops and peer-reviews are thought to potentiate the effect [25,38,53,55,56,61,64,65]. Similar to this and based on the idea of a less passive approach, one study showed that simply adding a signed commitment to the guideline information could decrease inappropriate prescriptions [35].

Furthermore, there is also mention of the important effect that specific tailoring of the intervention considering the conditions and needs of each situation, could enhance, even more, appropriate prescribing practices [25,31,32,40,41,55,58,64]. A simple example of how to apply this would be, when presenting the problem of antibiotic resistance, to use data relating the local impact [37].

Another issue regarding appropriate prescribing is related to diagnosis. Several studies also mention the importance of appropriate diagnosis in the reduction of antibiotic prescriptions [26,39]. As, for example, a more specific diagnosis will facilitate more accurate treatment prescription [48]. In many consultations, there is uncertainty on the diagnosis, and confirmation is rarely performed [29,30]. There are even cases where it is suspected that the antibiotic selection is done first, and then the diagnosis is determined in accordance with the selected therapy [27,31,65,66,67].

Even after employing these interventions, there is still a strong prevalence of inappropriate prescribing behavior in some cases. A commonly mentioned cause for prescribing antibiotics when they are not recommended is patient expectation [39,48,50,56,68,69,70]. One important step in improving interventions would then be to also include patients and parents, by educating them, there could be a reduction in the pressure felt by general physicians (GPs) to prescribe antibiotics [48]. Studies have already been done based on this premise, and show that combined intervention on GPs and patients was effective in decreasing antibiotics prescriptions [27,58].

Regarding the strict measures taken in order to reduce the rate of antibiotic prescriptions, that are reflected above, one might question if there could be an increase in complications due to the lack of therapy, however, studies have showed this to be highly unlikely, and would occur in only a very small percentage [30,45,71,72]. So, in this case, the positive impact reduction in antibiotic resistances surpasses the reduced possibility of inferences. Furthermore, one of the included studies actually conducted a follow-up of the patients in order to access whether they had been cured, felt an improvement, or felt the same, and results revealed there was no difference in clinical response between patients who received antibiotics and the ones that did not [58].

Within the studies regarding appropriateness, all of them focusing on pediatric had success in improving the quality of prescriptions. The four studies that somewhat failed focused on adults and elderly patients. These finding may suggest prescribers followed guidelines on choice of antibiotics in children and to a lesser degree, when treating adults, in accordance with studies developed in this area [73].

The results are, in general, positive, regardless of the continent, suggesting that guidelines implementation has success either in medium and high income countries in North America and Europe, as well as medium income countries in Asia. However, this cannot be generalized since there are a lack of studies designed in South America and Africa. PAL guidelines were implemented in low income countries with non-consensual results, mostly due to lack of adherence by the prescribers [52], which should be a main concern.

Within the scope of this review, another important outcome analyzed was the change in prescription costs. This is important since drug cost put an extreme strain in healthcare resources, in particular, but not restricted to low-income and developing countries, which can affect patients’ ability to fulfil their treatments and access care [37,52]. The way this correlates to this analysis is that unnecessary prescriptions are responsible for the increase in healthcare costs [74,75,76,77].

Actually, there have already been studies that show a reduction in cost as a result from a decrease in inappropriate prescribing habits [76,78,79]. The studies regarding this outcome were all related to primary care rather than hospital care. This aligns with the expectation for this study since primary care accounts for one-third of antibiotic prescriptions, and the majority of these prescriptions are for RTI [80]. In a great number of these primary care consultations, prescribing antibiotics does not have any health benefit for the patient [68,81,82]. More precisely, it has been shown that close to 40% of primary care consultations lead to antibiotics prescriptions, of which 20–50% are thought to be inappropriate [83,84,85]. In particular, for 30% of the consultations where a diagnosis of URTI was achieved, antibiotics are prescribed [82].

Focusing on the studies selected for this review, a strong correlation was described between an intervention aimed at reducing antibiotic prescriptions and a decrease in general drug costs [28,37,49,52,53,54]. This decrease seems to result from two different changes in the prescribing behavior. The first is an overall decrease in antibiotics prescriptions, which represents a costlier treatment [28,54]. The second, is the preference of inhaled medications rather than antimicrobial therapeutics, as these are responsible for a lower cost [28,49]. Despite some studies that failed to reach a significant reduction in cost, this method still seems promising [42]. An intervention which was shown to have a particular effect in decreasing cost was the implementation of PAL guidelines [28,52,53,54]. A next step in exploring this topic would be to do a more precise cost-effect analysis [54].

### Limitations

Inherent to any systematic review, ours suffers from the limitation of publication bias. The inclusion criteria allowed to cover a wide range of studies with different designs and, even though not all studies are randomized or controlled, we believe it is important to include all the studies in order to obtain the maximum information and a broader view of this issue. Besides, randomized and controlled trials showed positive results.

It is important to notice that the selected articles had quite a few issues, which can be understood for observing the quality assessment column in Table 1. Some of the more common issues are the lack of randomization [28,43,44,56], followed by the lack of blinding, which can result in an observed improvement not due to the intervention, but due to the fact participants know they are being evaluated [30,36,42,47,50,57]. Moreover, there were also studies where a control was not present, resulting in a reduced ability to access if the results are due to the intervention or external factors [38,45].

There is one particular characteristic of the studies which might affect the completely accurate representation of the results from the intervention, this is the method for data collection. As explained above, there are cases where data was obtained from medical records, from pharmacy records, or from insurance reports, each of these methods comes with its own set of limitations. In cases where medical records are used, whether the patient collects the prescription is not evaluated, so it does not represent consumption [44]. When data are collected from pharmacy records, this does not accurately represent the level of prescription as patients might have been prescribed antibiotics but did not collect them [41]. Finally, in the case of insurance reports, since prescriptions might not all be ensured, some might not be accounted for [25]. Furthermore, in all methods, one is still not able to confirm if patients completed the prescribed treatment correctly [41,44].

Possible exclusion of some foreign language trials has to be acknowledged. Although we cannot exclude that we have missed a few trials, we believe this would not have changed our conclusions. Despite these limitations, this systematic review summarizes and highlights with effectiveness the relevant studies, demonstrating the importance of guideline adherence in reducing antibiotic prescription and improving its quality.

## 4. Materials and Methods

### 4.1. Search Strategy and Inclusion Criteria

The PRISMA (Preferred Reporting Items for Systematic Reviews and Meta-Analyses) guidelines were followed [86] to perform this systematic review and the protocol for this review has been registered in the PROSPERO network (registration number: CRD42020172610).

In order to identify studies addressing the impact of guidelines adherence in the rate of antibiotic prescription for respiratory infections, the following search terms were used on the scientific databases MEDLINE-PubMED and EMBASE: guideline* AND respiratory* AND antibiotic* AND prescription. The articles considered for analysis were published before March 10 2020.

Research studies were eligible for inclusion if they were in accordance with the following selection criteria: (1) language (papers had to be published in English, Spanish, or Portuguese); (2) target population (prescribers); (3) intervention (exposure to guidelines for RTI); and (4) outcome measures (antibiotic prescription rates and/or quality for RTI before and after the intervention). To avoid selection bias in contexts not representative of the population in general, studies were excluded if they focused on specific patients or a specific pathology. Guidelines’ implementation included (1) educational interventions, where prescribers were introduced to the proper guidelines, in the form of (1.1) workshops, written sources (posters, pamphlets), or (1.2) decision support systems based on the guidelines; and (2) implementation of guidelines in a country or setting.

Insofar as study design of included studies was concerned, since this study aims to compare outcomes in two different time points, cross-sectional studies were not included as well as studies not including a pre and post intervention analysis (ex. comparison between an intervention group and the control group in the same point of time).

All titles and abstracts were independently screened by G.S, C.R, and I.O. The full text of potentially eligible articles was then screened independently to further evaluate its appropriateness for inclusion, validated by T.H and F.R in situations which there was no agreement.

### 4.2. Quality Assessment

For each individual study, risk of bias and quality assessment was conducted separately by D.G and I.O using the “quality assessment tool for quantitative studies”, from the Effective Public Health Practice Project [87]. Each study was rated regarding selection bias, study design, confounders, blinding, data collection methods, and withdrawals and dropouts. Based on the scores of these various components, each study was assigned an overall qualty of strong, moderate, or weak. In case of disagreement, T.H acted as referee to reach consensus.

### 4.3. Data-Extraction and Analysis

The included studies were summarized and organized in a table containing the author information, date of publication, country, study design, population, source data, type of guideline and outcomes. The information for data extraction was retrieved from the articles exactly as described in the study, and when these characteristics were not specified in the text, it was considered non defined (n.d). Data were extracted by G.S and C.R and their assessments were compared. In case of disagreement, F.R acted as referee to reach consensus.

The source of data was classified as: (1) primary care; (2) hospital care; (3) nursing home; (4) medical training facility.

The guideline’s implementation was classified according to whether it included a broader intervention or if it was an international/national/local guideline implementation focused study. The period during which outcomes were measured (baseline, intervention period, and follow-up) was also included.

The outcomes extracted from studies consisted of changes before and after the guideline’s implementation in: (1) rates of antibiotics prescribed for patients with RTI; (2) rates of guideline-recommended (appropriate) antibiotics prescriptions for patients with RTI. As secondary outcome changes in treatment, related costs were also extracted. This was evaluated either by looking into antibiotics prescription cost or by looking into a general setting where prescriptions of non-antibiotics are also considered. Study results were classified as: positive (+), if changes in outcomes measured were statistically significant; partially positive (±), if reported as positive for some variables and negative for others; and negative (−), if no significant changes were observed.

## 5. Conclusions

Published studies varied widely in terms of study design, implementation period, and type of patient. Nevertheless, it can be concluded from this review that guidelines implementation in order to improve prescriber’s adherence can be effective in reducing antibiotic prescriptions for RTIs and improve its quality. These interventions have also been shown to be potentially effective in reducing treatment costs related to RTIs.

## Figures and Tables

**Table 1:** Summary of the characteristics of the included studies.

Author (year)	Country ^a^	SP ^b^	Setting ^c^	SS ^d^	P/CC ^e^	TP ^f^	SD ^g^	Source of Data ^h^	TG ^i^	Quality Assesment
[57] Stewart et al. (2000)	CN	P, OH, PA	PC	15	URTI, LRTI	n.d	Before and after controlled trial	PH, HI	Nat	Weak
[25] Wilson et al. (2003)	AU	P	HC	54 Pre, 40 Post	ARTI	Pe	RCT	HI	Loc	Weak
[26] Thamlikitkul and Apisitwittaya (2003)	TH	P	HC	12 + 837 C Pre, 774 C Post	URTI	A	n.d	MR	Loc	Weak
[37] Pagaiya and Garner (2005)	TH	Oh	PC	18	ARTI	Pe	RCT	MR	Nat	Moderate
[48] Razon et al. (2005)	IL	P	PC	27 + 4580 C Pre, 4364 C Post	URTI	Pe 3 months to 18 years	Multicenter before and after study	MR	Int	Moderate
[51] Rautakorpi et al. (2006)	FI	P	PC	453 Pre, 709 Post + 7774 C Pre, 10322 C Post	RTI, UTI	Pop	n.d	MRS	Nat	Weak
[52] Shrestha et al. (2006)	NP	P, Oh	PC	238 C Pre, 168 C Post	Asthma, COPD, PNA	A (34 to 70 years)	cRCT	MR	Nat	Moderate
[53] Camacho et al. (2007)	BO	P	PC	78 + 1033 C Pre, 1154 C Post	URTI, LRTI, CRD	Pop (9 to 58 years)	n.d	MRS	Nat	Weak
[54] Brimkulov et al. (2009)	KG	P	PC	86 + 893 C Pre, 992 C Pre	URTI, LRTI, CRD	Pop (5 to 49 years)	n.d	MRS	Nat	Moderate
[55] Smeets et al. (2009)	NL	P	PC	382	RTI	A (40 to 55 years)	Controlled before and after trial	HI	Nat	Weak
[56] Reyes-Morales et al. (2009)	MX	P	PC	106	ARTI	Pop (1 to 85 years)	NRC pre post T	I, MR	Loc	Weak
[28] Me’emary et al. (2009)	SAR	P	PC	76 + 1806 C Pre, 75+1099 C Post	URTI, LRTI, CRD	Pop (5 to 42 years)	n.d	MRS	Loc	Weak
[27] Bjerrum et al. (2011)	AR, DK, LT, RU, ES, SE	P	PC	440 + 24436 C Pre, 22575 C Post	URTI, LRTI	Pop (3 to 62 years)	n.d	MRS	Int	Weak
[29] Angoulvant et al. (2011)	FR	P	PC, HC	11260 C Pre, 15505 C Post	ARTI	Pe (<18 years)	Retrospective	MR	Nat	Weak
[30] Dommergues and Hentgen (2012)	FR	P	HC	400 GP + 435 S	URTI	Pe (<18 years)	Observational	MRS	Nat	Moderate
[31] Gerber et al. (2013)	USA	P	PC	170 Pre; 162 Post	ARTI	Pe (1 to 10 years)	cRCT	MR	Nat	Weak
[32] Grover et al. (2013)	USA	P, Oh	PC	17 + 241 C	ARTI	Pop	n.d	MRS	Nat	Weak
[33] Gjelstad et al. (2013)	NO	P	PC	489 Pre, 382 Post	ARTI	Pop	cRCT	MR	Nat	Weak
[34] Angoulvant et al. (2013)	FR	P	PC, HC	149018 C	ARTI, URTI	Pe (<18 years)	Prospective observational	MR	Nat	Weak
[58] Boonyasiri and Thamlikitkul (2014)	TH	P, OH	AC	23637 C Pre + 1241 C Pos	URTI,	Pop (>2 years)	n.d	MRS	Nat	Weak
[35] Meeker et al. (2014)	USA	P, PA	PC	14	ARTI	A	RCT	MR	n.d	Weak
[36] Zimmerman et al. (2014)	USA	P, Oh, PA - (NH)	NH	n.d	RTI, UTI, SSTI	A, EP (>18 years)	Q-E	MRS	Nat	Weak
[38] Urrusuno et al. (2014)	ES	P	PC	703 C Pre; 884 C Post	RTI, UTI, SSTI	Pop	Q-E	PR, MR	Loc	Weak
[39] van Buul et al. (2015)	NL	P, Oh (NH)	NH	(10) 707C Pre; 552C Post	RTI, UTI, SSTI	EP (>43 years)	Q-E	MRS	Nat	Weak
[40] Hingorani et al. (2015)	USA	P, Oh	HC	28	ARTI, URTI	Pop	n.d	MR	Nat	Weak
[41] van der Velden et al. (2015)	NL	P	PC	165 Pre; 163 Post	RTI	A	cRCT	PR	Nat	Weak
[42] Ferrat et al. (2016)	FR	P	PC	203 Pre, 168 Post	RTI	Pop	RCT	HI	Nat	Weak
[43] Dyrkorn et al. (2016)	NO	P	PC	53 + 1271 C Pre, 1212 C Post	ARTI	Pop	nRCT	MR	Nat	Weak
[44] Magin et al. (2017)	AU	P	MTF	528 Pre, 213 Post	URTI	Pop	nRCT	MR	Nat	Weak
[45] Ouldali et al. (2017)	FR	P	PC	61612 C Pre, 134450 C Post	ARTI	Pe (<18 years)	Q-E, ITS	MR	Nat	Weak
[47] Molero et al. (2018)	ES	P	PC	224 Pre, 238 Post	RTI	Pop	n.d	MRS	Nat	Weak
[46] Sloane et al. (2019)	USA	P, Oh, PA - (NH)	NH	(27)	RTI, UTI, SSTI	EP (over 85)	quality improvement trial with two arms	NHR	n.d	Weak
[49] Wei et al. (2019)	CN	P, PA	PC	2800 C Pre, 8769 C Post	URTI	Pe (2 to 14 years)	cRCT	MR	n.d	Weak
[50] Aoybamroong et al. (2019)	TH	P	MTF	2553 C Pre, 2935 C Post	ARTI	Pe	Prospective before–after study	MR	Loc	Weak

^a^ Country: AU, Australia; TH, Thailand; FI, Finland; NP, Nepal; SW, Switzerland; NL, Netherlands; MX, Mexico; SAR, Syrian Arab Republic; AR, Argentina, DK, Denmark; LT, Lithuania; RU, Russia; ES, Spain; SE, Sweden; FR, France; USA, United States of America; NO, Norway; CN, China; IL, Israel; BO, Bolivia; KG, Kyrgyzstan; CN - Canada. ^b^ Study population: P, physicians; Pt, physician trainees; PA, patients; Oh, other healthcare providers. ^c^ Setting: PC, primary care; HC, hospital care; AC, ambulatory care; NH, Nursing home; MTF, medical training facility. ^d^ Sample size: Pre, pre-intervention/study beginning; Post, post-intervention/study end; C, consultations. ^e^ Pathology/clinical condition: RTI, respiratory tract infection; ARTI, acute respiratory tract infection; URTI, upper respiratory tract infection; LRTI, lower respiratory tract infection; CRD, chronic respiratory disease; COPD, chronic obstructive pulmonary disease; PNA, pneumonia. ^f^ Type of patient: Pe, pediatric; A, adult; EP, elderly persons; Pop, all population. ^g^ Study design: RCT, randomized controlled trial; nRCT, non-randomized controlled trial; nRnCT, non-randomized non-controlled trial; cRCT, cluster-randomized controlled trial; cRnCT, cluster-randomized non-controlled trial; RCS, retrospective cohort study; PCS, prospective cohort study; Q-E, quasi-experimental; ITS, interrupted time series. ^h^ Source of data: PH, pharmacists claims; HI, health insurance; MR, medical records; MRS, medical records obtained from surveys; I, interviews; PR, pharmacy records; NHR, nursing home records.^i^ Type of guideline: Int, international; Nat, national; Loc, locally prepared; NA, non applicable; n.d, not determined.

**Table 2:** Summary of interventions of the included studies.

Author (year)	Implementation ^a^	Implementation Period			Results ^c^
		Baseline	Post-Implementation	Follow-up	
[57] Stewart et al. (2000)	I	6 months (Oct 1995–Mar 1996)	6 months (Oct 1996–Mar 1997)	n.d	AP(+)
[25] Wilson et al. (2003)	G	1 year	2 year	n.d	QA (+) ^1^
[26] Thamlikitkul and Apisitwittaya (2003)	G	3 months	3months	n.d	QA (+); AP(+) ^2^
[37] Pagaiya and Garner (2005)	G	n.d	6 months	n.d	QA (+); CP(+)
[48] Razon et al. (2005)	G	4 months (Nov 1999–Feb 2000)	4 months 4 months (Nov 2000–Feb 2001)	n.d	QA (+)
[51] Rautakorpi et al. (2006)	I ^1^	1 week (Nov 1998)	1 week (Nov 2001)	1 week (Nov 2001)	AP (±) ^3^
[52] Shrestha et al. (2006)	G ^2^	4months (Feb–May 2002)	4months (Oct 2002–Jan 2003)	n.d	AP (-), QA (-), CP (-)
[53] Camacho et al. (2007)	G ^2^	5 days (July)	5 days (Sept)	28 days	QA (-), CP (+)
[54] Brimkulov et al. (2009)	G ^2^	1 week (Nov)	1 week (Dec)	1 month	QA (+), CP (+)
[55] Smeets et al. (2009)	I ^3^	6 months	6 months	6 months	QA (-), AP (-)
[56] Reyes-Morales et al. (2009)	I ^3^	n.d	3 months	n.d	AP (+)
[28] Me’emary et al. (2009)	G ^2^	5 days (Dec)	5days (Jan)	30 days	QA (+), CP (+)
[27] Bjerrum et al. (2011)	I ^4^	3 weeks (winter 2008)	3 weeks (winter 2009)	n.d	QA (±) ^4^, AP (+)
[29] Angoulvant et al. (2011)	G	1 year	3 years	n.d	QA (+)
[30] Dommergues and Hentgen (2012)	G	5 years	5 years	n.d	QA (+)
[31] Gerber et al. (2013)	G	20 months	12 months	n.d	AP (+) ^5^
[32] Grover et al. (2013)	I ^5^	n.d	2 months and 2 days	n.d	QA (+)
[33] Gjelstad et al. (2013)	I ^3^	3 months	1 year	1 year	QA(+); AP(+) ^6^
[34] Angoulvant et al. (2013)	G	2 years	1 year	n.d	AP (+) ^7^; QA (+)
[58] Boonyasiri and Thamlikitkul (2014)	I ^3^	4 months	4 months	n.d	QA (+)
[35] Meeker et al. (2014)	G ^6^	9 months	3 months	n.d	AP (+)
[36] Zimmerman et al. (2014)	I ^5^	3 months	6 months	n.d	QA (+)
[38] Urrusuno et al. (2014)	G	1 year	1 year	n.d	AP (+)
[39] van Buul et al. (2015)	I	1 year (July 2010–June 2011)	18 months (Jan–sept 2012 and Jan–sept 2013)	n.d	AP (-)
[40] Hingorani et al. (2015)	G	4 years	5 months	n.d	AP (+)
[41] van der Velden et al. (2015)	I ^3^	1 year	1 year	1 year	AP (+), QA (+)
[42] Ferrat et al. (2016)	G	3 months	3 months per year (5 years)	n.d	QA (+), CP (-)
[43] Dyrkorn et al. (2016)	G	1 year	1 year	n.d	AP (+)
[44] Magin et al. (2017)	I ^3^	2 years	1 year	n.d	QA(±) ^8^
[45] Ouldali et al. (2017)	G	2 years	3 years	n.d	AP (+), QA (+)
[47] Molero et al. (2018)	I ^4^	15 days (2008)	15 days (2009)	15 days (2015)	AP (+)
[46] Sloane et al. (2019)	I ^5^	4 months	18 months	6 months	QA (-)
[49] Wei et al. (2019)	I ^7^	3 months	6 months	12 months	AP (+), QA (+), CP (+)
[50] Aoybamroong et al. (2019)	G	6 months	6 months	n.d	AP (+)

^a^ I—broader intervention including guideline implementation; G—guidelines implementation as main focus. ^1^ MIKSTRA program; ^2^ Practical Approach to Lung Health (PAL) guidelines; ^3^ multifaceted intervention; ^4^ Health Alliance for prudent antibiotic prescribing in patients with respiratory tract infections (HAPPY AUDIT) a multifaceted intervention program; ^5^ quality improvement program; ^6^ guideline nudging; ^7^ antibiotic stewardship program. ^c^ QA—rates and quantity of antibiotics prescribed for patients with RTI; AP—rates of appropriate antibiotics prescribed for patients with RTI; CP—differences in cost prescriptions. ^1^ The average yearly prescribing decreased significantly in the intensive intervention group and increased in the moderate intervention group, (*p* = 0.026); ^2^ There was a significant reduction in use of amoxicillin, co-trimoxazole, roxithromycin, and doxycycline; and penicillin V was prescribed significantly more often; ^3^ Use of first-line antibiotics increased for all infections, and the change was significant for sinusitis (*P* < 0.001), acute bronchitis (*P* < 0.015); ^4^ A significant reduction in the antibiotic prescribing rate was found in the Baltic countries and Hispano-America, while no significant change was seen in the Nordic countries; ^5^ Broad-spectrum antibiotic prescribing considered off guidelines, and significantly decreased; ^6^ less use of non-penicillin V antibiotics; ^7^ The percentage of amoxicillin prescriptions increased dramatically during the study; The percentages of amoxicillin-clavulanate and cefpodoxime prescriptions decreased; ^8^ Reduced antibiotic prescribing for acute bronchitis/bronchiolitis but not for URTIs.
